# Machine learning provides novel neurophysiological features that predict performance to inhibit automated responses

**DOI:** 10.1038/s41598-018-34727-7

**Published:** 2018-11-02

**Authors:** Amirali Vahid, Moritz Mückschel, Andres Neuhaus, Ann-Kathrin Stock, Christian Beste

**Affiliations:** 1 Cognitive Neurophysiology, Department of Child and Adolescent Psychiatry, Faculty of Medicine of the TU Dresden, Saxony, Germany; 2 Department of Psychiatry, Charite University Hospital Berlin, Berlin, Germany

## Abstract

Neurophysiological features like event-related potentials (ERPs) have long been used to identify different cognitive sub-processes that may contribute to task performance. It has however remained unclear whether “classical” ERPs are truly the best reflection or even causal to observable variations in behavior. Here, we used a data-driven strategy to extract features from neurophysiological data of n = 240 healthy young individuals who performed a Go/Nogo task and used machine learning methods in combination with source localization to identify the best predictors of inter-individual performance variations. Both Nogo-N2 and Nogo-P3 yielded predictions close to chance level, but a feature in between those two processes, associated with motor cortex activity (BA4), predicted group membership with up to ~68%. We further found two Nogo-associated features in the theta and alpha bands, that predicted behavioral performance with up to ~78%. Notably, the theta band feature contributed most to the prediction and occurred at the same time as the predictive ERP feature. Our approach provides a rigorous test for established neurophysiological correlates of response inhibition and suggests that other processes, which occur in between the Nogo-N2 and P3, might be of equal, if not even greater, importance.

## Introduction

Neurophysiological features such as event-related potentials (ERPs) correlate with behavioral performance in a wide range of different tasks and have therefore been used to identify different cognitive sub-processes that contribute to task performance. Yet still, it has remained largely unclear whether “classical” ERPs are truly the best reflection or even causal to observable variations in behavior. While there is a lot of literature that helps to interpret one’s findings^1^, most of the investigated differences / ERPs are defined by minima, maxima, or pre-defined time windows in the course of the neurophysiological times series. This excludes a wealth of data “in between” the typically quantified neurophysiological features. This bias is especially dramatic given that any given EEG signal is composed of different signals, which do not only vary in latency, but also stem from different neuronal generators within the human brain^2–6^. To address this issue, many recent studies have applied complex signal decomposition approaches^2,7–9^, but even those approaches usually limit themselves to minima and maxima of the different identified components and have therefore also remained correlational.

Fortunately, the recent rise in machine learning approaches and methods has equipped us with a new and powerful tool that can expediently and objectively identify differences in the entire EEG signal. It may furthermore help to identify neurophysiological features that allow to predict behavioral performance, instead of just correlating with it. In fact, it has recently been argued that machine learning approaches may foster theoretical achievements and improve our understanding of the brain dynamics underlying cognitive functions^10^. To achieve this objective, it is important to move beyond ERP components in EEG research^10^. While a few studies have used ERP data to predict behavioral performance with the help of support vector machines (SVMs)^11–13^, such approaches are still not widely used. Moreover, these approaches are often strongly influenced by assumptions about the functional relevance of specific ERP-components and therefore based on a biased selection of potential features^11–13^. This means that neurophysiological features currently used for behavioral predictions with SVM approaches have often been pre-selected to represent certain neurophysiological characteristics, such as specific ERP peaks for which correlational differences have previously been observed. Even though these approaches can predict (i.e. classify) the difference between experimental conditions or correctly and incorrectly answered trials above chance level, classification performance is often not very high^12,13^. For a critical discussion of this issue, see^14^. In this context, please note that throughout the entire manuscript, the term prediction will be strictly used in the context provided by machine learning approaches. This is, how well can individuals with “good” or “bad” behavioral performance be distinguished with the help of a given neurophysiological feature?

One possible reason for the rather low predictability may relate to the a-priori and literature-based selection of ERP features (as also done in a previous publication of our work group^13^,). While the pre-existing literature may help to interpret findings, it might also impede the discovery of novel neurophysiological features, especially “outside” of traditional ERP time windows and / or topographies. Yet, those might still be important to consider when trying to obtain a more comprehensive understanding of neural mechanisms that allow to predict behavioral performance.

To tackle this issue, we chose to investigate motor inhibition (i.e. the capability to interrupt an automated response tendency), as it has already been investigated for decades and is known to reliably produce two different ERPs that closely correlate with behavioral performance, i.e. the frontal-midline Nogo-N2 and the Nogo-P3^2^. While the Nogo-N2 ERP component is assumed to represent pre-motor processes like conflict monitoring, the Nogo-P3 component may reflect the inhibitory process itself or an evaluation process thereof ^2,15–18^.

Yet still, ERPs reflect a mixture of different neuronal sources as well as oscillations from various frequency bands^19,20^, but not all of these frequencies are equally important for the investigated cognitive process^21^. This might have important implications for the prediction of behavioral performance. In this context, oscillations in the theta frequency band could be particularly important as they are considered to play a pivotal role in cognitive control and executive functions^22–26^, including the ability to inhibit prepotent motor responses^27,28^. In line with this, it has been shown that response inhibition-related processes are associated with oscillations in the theta frequency band^2,15,29–32^. Yet, the alpha frequency band may also be relevant for inhibitory control^33–37^ because oscillations in this frequency band seem to coordinate top-down control processes^34,38^. Furthermore, alpha oscillations may reflect inhibitory mechanisms which control access to task-relevant information^36^; i.e. updating processes of internal task-sets being used^35^. Against this background, it is conceivable that time-frequency (TF) decomposed data may provide a better prediction of response inhibition performance than regular ERP data, as the latter also contains signals from less relevant frequency bands, like beta oscillations. Until now it has however not been directly tested whether theta, beta, or alpha frequency oscillations allow for a better prediction of behavioral response inhibition performance than regular ERPs, which comprise a combination of all of these oscillations. If we found that one of these frequency bands was most predictive for behavioral performance, this would suggest that current views and theoretical conceptions about the importance of the other frequency bands, that are considered to play a role in response inhibition, may need to be refined. Yet, it is also possible that a combination of features (i.e. different frequency bands) may show high predictability of motor response inhibition performance. This would suggest that current views about the frequency architecture underlying motor response inhibition processes, which still often stress the importance of either theta or alpha oscillations, are too simplified

In the current study, we employed a data-driven feature extraction strategy in combination with support vector machine (SVM) classification approaches to predict inter-individual (i.e. relative, not absolute or categorial) differences in response inhibition performance from objectively identified neurophysiological features, as compared to regular Nogo-N2 and P3 ERPs. The main reason for choosing this approach is that these “classical” inhibition-related ERPs are usually also used to depict inter-individual differences in inhibitory performance. Specifically, we examined which time-frequency features as well as features in the time domain (i.e. ERPs) best predict response inhibition performance, as defined by group membership (good vs. bad performers). For the ERP data, this was combined with source localization methods to examine which functional neuroanatomical structures are associated with these features. Our main reason for using classification instead of regression here is that the brain is a nonlinear system. Consequently, the assumption that there is a linear relation between EEG signal and behavior is not very likely. Non-linear regression methods however require a-priori knowledge about the degree of polynomial function and the nature of the non-linear relationship between the predictor(s) and the dependent variable(s). This is however not (yet) the case in this field of research. Therefore, the primary goal of this work is to find a nexus between neurophysiology and behavior without any prior assumption. Lastly, results from regression analyses are more affected by outliers than SVM-based classification methods. In order to form two equally large groups that differ in performance, we formed a behavioral ratio that accounts for the relation of speed and accuracy by dividing the number of correct inhibitions in the Nogo condition by the response time in Go trials. The reasoning behind this was that previous analyses showed that individuals with low false alarm often respond more slowly to Go trials than individuals with high false alarm rates^13^. Given that slow responses leave more time to carry out a motor inhibition^39^, not accounting for the speed-accuracy-tradeoff would have confounded our analyses. As better performance may hence be reflected by better accuracy (i.e. larger accuracy values) and/or faster responses (i.e. smaller response time values), the ratio we used becomes larger as behavioral performance improves. Based on this, participants with relatively small ratio values may be labeled “bad performers” while participants with relatively large values may be labeled “good performers”. We hence divided the sample into an equal number of good and bad performers (please see methods section for details).

In addition to providing a rigorous evaluation of neurophysiological processes that are important for response inhibition, our data-driven approach also confers another advantage, because it may help to generate new hypotheses^40^. It is therefore possible to derive new, testable hypotheses about the neurophysiological processes and associated functional neuroanatomical structures that determine response inhibition performance. If we found features that complement the currently investigated Nogo-N2 and P3, this would extend or challenge current thinking about the mechanisms underlying the behavioral outcome of response inhibition.

## Results

### Behavioral data and group differences

For the descriptive statistics, the mean and standard error of the mean are given. On average, participants responded correctly in 98.75% (±0.10) of Go trials (hits). The average reaction time (RT) was 348 ms (±2.73). About 12.12% (±0.69) of Nogo trials were not correctly inhibited (false alarms). The mean quotient used for the estimation of behavioral performance (correct inhibition rate divided by mean hit RT) was 0.254 (±0.002; Supplementary Fig. 3). Importantly, this transformation accounts for the speed-accuracy tradeoff: good performance is characterized by relatively lower values (due to lower false alarm rates and/or faster Go responses) while poor or “bad” performance is characterized by relatively larger values (due to higher false alarm rates and/or slower Go responses). We used this criterion to form two equally large groups (“good” vs. “bad” performers) using a median split (median = 0.257). Despite the fact that this criterion is relative, rather than absolute or categorial (like the distinction between false and correct trials), it is the most appropriate criterion for our research question, i.e. the mechanisms underlying inter-individual performance variations. The reason for this is that the level/quality of an individual’s task performance can only be assessed in relative comparison to others’ performance the same task.

As evidenced by a Mann-Whitney-U test, the two groups significantly differed with respect to their behavioral ratio values (p < 0.001). On average, the low performance group had a ratio of 0.2313 ± 0.0018 while the high performance group had a mean behavioral ratio of 0.2757 ± 0.0012. The groups furthermore differed with respect to Go hit RTs (low = 365.1 ms ± 3.56; high = 331.9 ms ± 0.49) and Nogo FAs (low = 15.74% ± 0.89; high = 8.56% ± 1.91), when tested separately (both p < 0.001). The two performance groups did however not differ with respect to sex (χ² = 0.817; p = 0.366), age (p = 0.283), handedness as assessed with the Edinburgh Handedness Inventory (p = 0.432)^41^, or depression symptoms as assessed with Beck’s depression inventory (p = 0.959)^42^.

### Standard ERP data analysis

**Figure 1:**
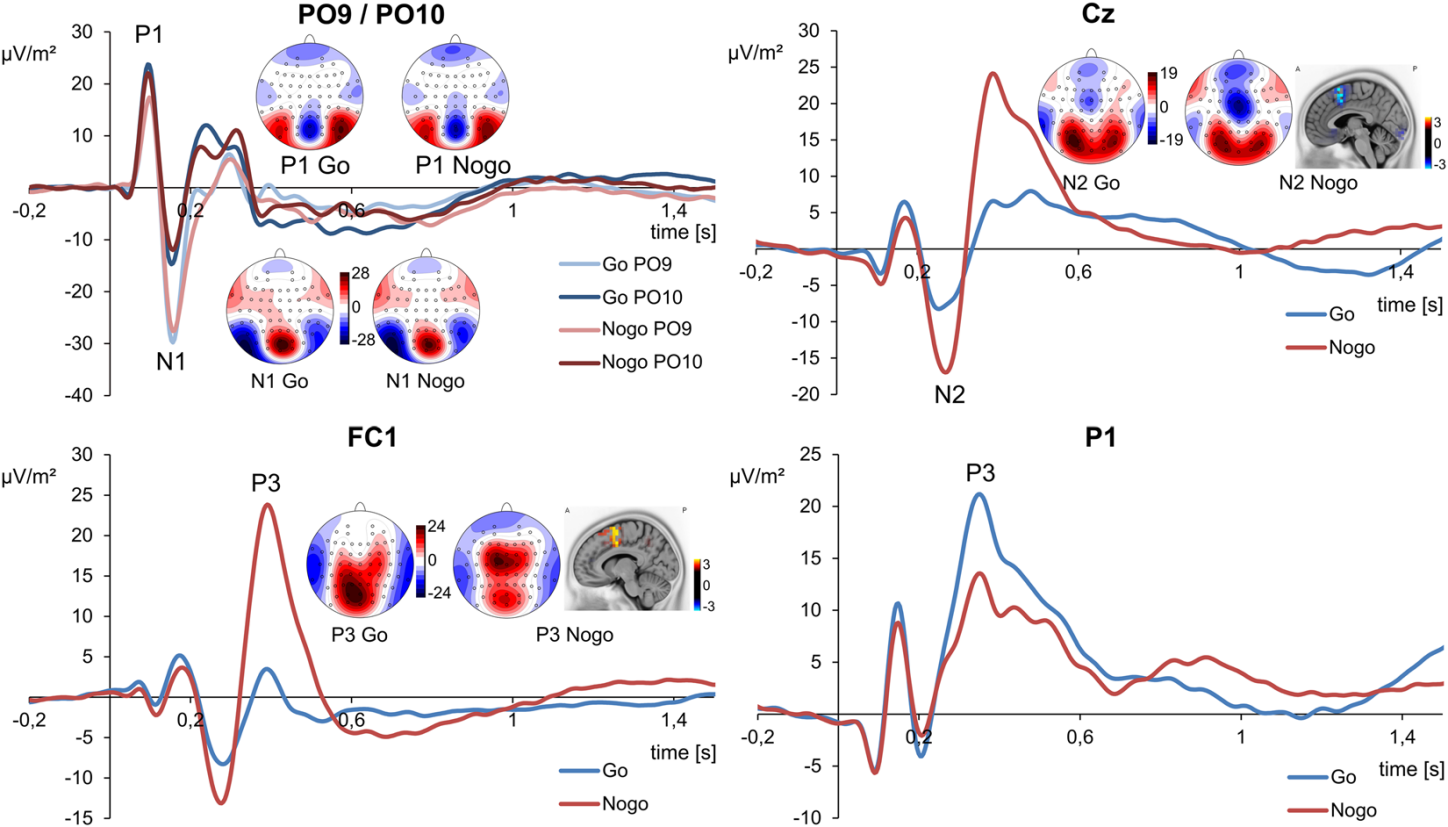
Event-related potential (ERP) components on Go and Nogo stimuli presentations. Plots are given for electrodes PO9/PO10 depicting P1 and N1 ERP, electrode Cz for N2, electrode FC1 for Nogo-P3 as well as electrode P1 for Go-P3. ERPs on Go stimuli are shown in blue, ERPs on Nogo stimuli are shown in red. The scalp topography plots reveal the distribution of voltages at the time point of the peak maximum of each ERP component. Time point zero denotes the time point of stimulus delivery. For N2 and P3 ERP, the results of sLORETA source estimation are shown.

The P1, N1, N2 and P3 ERP-components in Go and Nogo trials are shown in Fig. 1. For a figure of ERP-components by group, please refer to the Supplementary Material (Supplementary Fig. 1). In any of the analyses described below, the factors handedness and depressive symptoms did not change the pattern of results (all F < 0.67; p > 0.510).

For the P1 ERP amplitude, the repeated-measures ANOVA revealed a significant main effect of condition (F(1, 238) = 10.73; p = 0.001; η²_p_ = 0.04). The P1 ERP amplitudes were larger in Go trials (19.45 µV/m² ± 0.74) than in Nogo trials (18.67 µV/m² ± 0.75). The significant main effect of electrodes (F(1, 239) = 13.46; p < 0.001; η²_p_ = 0.05) suggested that the P1 amplitude was larger at electrode PO10 (21.66 µV/m² ± 1.14) as compared to electrode PO9 (16.47 µV/m² ± 0.88). Additionally, there was a significant interaction effect of condition x electrodes (F(1, 238) = 9.22; p = 0.003; η²_p_ = 0.04). As shown by a post-hoc t-test (t(239) = −3.04; p = 0.003), the P1 amplitude difference between Go and Nogo trials was stronger for electrode PO10 (1.63 µV/m² ± 0.36) than for PO9 (−0.06 µV/m² ± 0.38). All other effects including the main effect of group were not significant (all F < 0.67; p > 0.420).

For the N1 amplitudes, a significant main effect of condition (F(1, 238) = 21.43; p < 0.001; η² = 0.08) showed that the N1 amplitude was more negative in Nogo trials (−12.62 µV/m² ± 0.95) than in Go trials (−10.82 µV/m² ± 1.00). The significant main effect of electrodes (F(1, 238) = 86.65; p < 0.001; η²_p_ = 0.27) indicated that N1 amplitudes were more negative at PO9 (−18.15 µV/m² ± 1.20) than at PO10 (−5.29 µV/m² ± 1.16). The interaction effect of condition x electrodes was significant (F(1, 238) = 18.20; p < 0.001; η²_p_ = 0.07). Subsequent t-test (t(239) = −4.27; p < 0.001) showed that the N1 amplitude difference of Go and Nogo trials was larger for electrode PO9 (3.50 ± 0.61) than for electrode PO10 (1.00 ± 0.50). Additionally, there was a significant interaction effect of condition x group (F(1, 238) = 7.27; p = 0.008; η²_p_ = 0.03). As shown by a significant post-hoc t-test (t(210,36) = −2.70; p = 0.008), the N1 amplitude difference between Go and Nogo trials was larger in the high performance group (5.70 ± 1.28) than in the low performance group (1.50 ± 0.88). The main effect of group as well as all other interaction effects were not significant (all F < 1.34; p > 0.249).

For the N2 amplitudes, the ANOVA showed a significant main effect of condition (F(1, 238) = 147.76; p < 0.001; η²_p_ = 0.38), indicating that the N2 was more negative in Nogo trials (−16.22 µV/m² ± 0.98) than in Go trials (−7.91 ± 0.80). The sLORETA analysis showed that these condition differences in Nogo-N2 amplitudes were associated with activation modulations in the medial frontal cortex and the superior frontal gyrus (BA8) in particular. The main effect of group as well as the interaction effect were not significant (all F < 1.97; p > 0.162).

For the P3 amplitudes at frontal-central electrode FC1 (i.e. the Nogo-P3), a significant main effect of condition (F(1, 238) = 749.51; p < 0.001; η²_p_ = 0.76) was found. This indicated that the frontal-central P3 amplitude was larger in Nogo trials (23.11 µV/m² ± 1.04), as compared to Go trials (3.27 µV/m² ± 0.73). The sLORETA analysis showed that these condition differences in P3 amplitudes were associated with modulation of activity in the medial frontal cortex including the medial frontal gyrus (BA6) and the anterior cingulate cortex (ACC, BA24). As indicated by a significant main effect of group (F(1, 238) = 10.59; p = 0.001; η²_p_ = 0.04), the frontal-central P3 amplitudes were larger in the high performance group (15.86 ± 1.16) than in the low performance group (10.51 ± 1.16). Additionally, there was a significant interaction effect condition x group (F(1, 238) = 10.62; p = 0.001; η²_p_ = 0.04). To further analyze this interaction effect, the difference of frontal-central P3 amplitudes of Nogo and Go was calculated. A significant post-hoc t-test (t(238) = 3.26; p = 0.002) showed that the frontal-central P3 amplitude difference was larger in the high performance group (22.20 ± 0.94) than in the low performance group (17.47 ± 1.10). For the P3 amplitudes at posterior electrode P1 (i.e. the Go-P3), the ANOVA revealed a significant main effect of condition (F(1, 238) = 197.76; p < 0.001; η²_p_ = 0.45). This indicated that posterior P3 amplitudes were larger in Go trials (17.47 ± 0.68) in comparison to Nogo trials (10.47 ± 0.70). There was also a main effect of group (F(1, 238) = 5.88; p = 0.016; η²_p_ = 0.02). Posterior P3 amplitudes were larger in the high performance group (15.52 ± 0.91) than in the low performance group (12.42 ± 0.91). Again, there was a significant interaction effect condition x group (F(1, 238) = 5.34; p = 0.02; η²_p_ = 0.02). For further analysis of this interaction effect the difference of Nogo and Go P3 amplitudes trials was computed. A significant post-hoc t-test (t(238) = −2.31; p = 0.044) showed that the difference of posterior P3 amplitudes between Nogo and Go trials was larger for the high performance group (−8.15 ± 0.75) than for the low performance group (−5.85 ± 0.65).

Taken together, the analysis of standard ERP-components revealed the usual pattern of results and that the topography of the Nogo-N2 and Nogo-P3 was centered around fronto-central electrodes^2^.

### Time frequency analysis

As can be seen in Supplemental Fig. 2 for Nogo trials (right), a power increase is evident in the theta frequency band about 200 to 350 ms post stimulus with a fronto-central topography. To analyze whether this power increase differed between conditions as well as between groups, a repeated measures ANOVA (condition x groups) was calculated. Therefore, the mean theta oscillatory power was determined in the frequency range of 5 to 6 Hz, in the time window of 240 to 320 ms post stimulus for pooled electrodes FCz and Cz. There was a significant main effect of condition (F1,238) = 192.51; p < 0.001; η²_p_ = 0.45). Theta power was larger for Nogo trials (87.35 ± 4.70) than for Go trials (33.00 ± 1.55). All other effects were not significant (all F < 2.84, p > 0.093).

**Figure 2:**
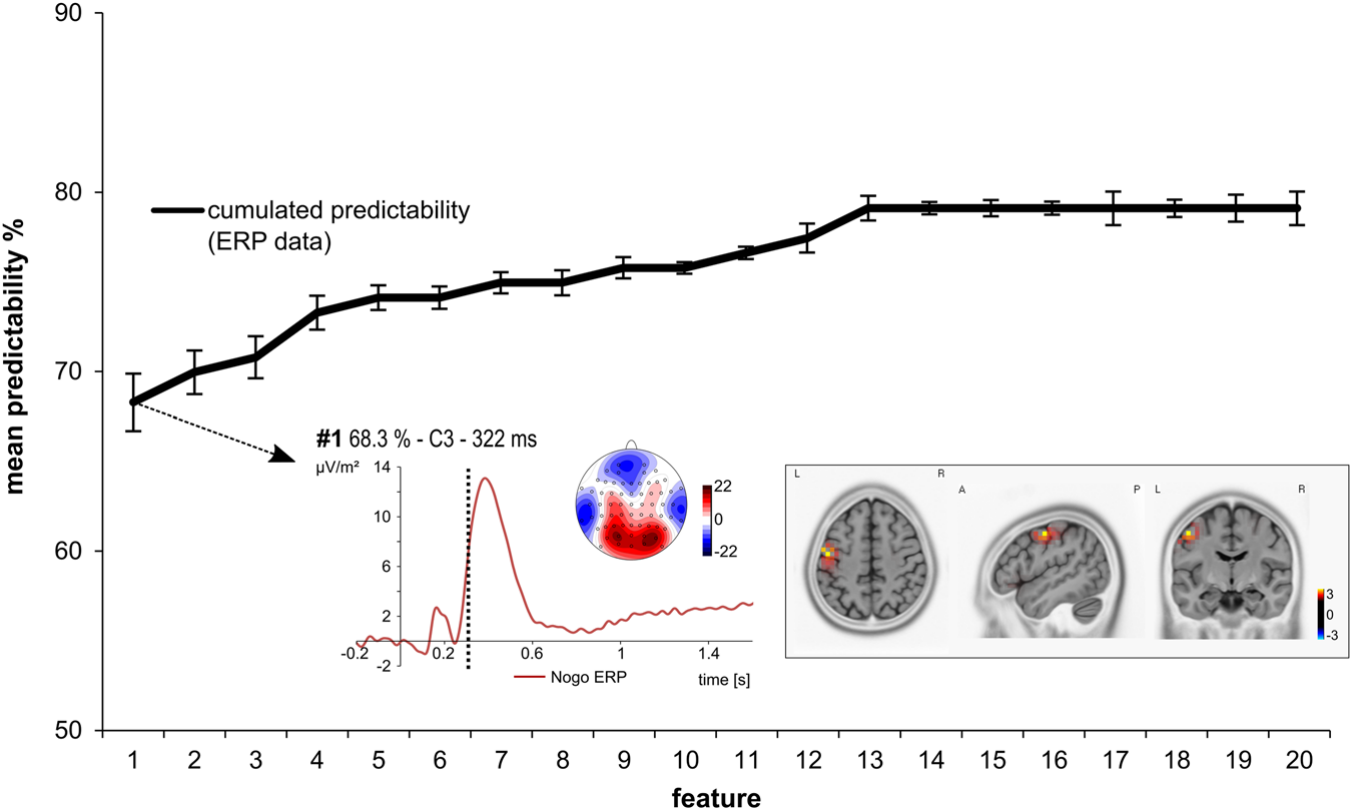
Results from the classification analysis using the time domain ERP data. The mean predictability is given depending on the number of features. The black curve in the figure shows the cumulative mean predictability. The error bars represent the 99% confidence level bounds. As can be seen the confidence bounds were overlapping for the first and the second feature. The ERP-curve of the first feature is also shown. The dashed vertical line in the plot shows the time point (feature) at electrode C3. The scalp topography plot reveals the distribution of voltages for this feature. The sLORETA plots (corrected for multiple comparisons using SnPM) show the source of the signal at the time point of the feature.

### Correlation analyses

Linear regression analyses were performed for the above-mentioned ERP components to examine correlations between ERP data and the behavioral data. This was done for each group separately and also including the factor “group” in the regression model (inclusion method). There were no linear correlations between any ERP parameter and the behavioral data (all r < 0.2; p > 0.3; all F < 0.62; p > 0.4).

### Machine learning analysis

As outlined in the methods section we used the k-fold method (k = 10) to evaluate the predictability of behavioral performance (as assessed via membership in either the ‘good performer’ or ‘bad performer’ group) using ERP and TF-decomposed data. This means that for each extracted feature, there were 10 varying estimations of the predictability of behavioral performance. Using the data from the k = 10 estimations we calculated the 99% confidence bounds for each feature. A significant difference is indicated by no overlap between the calculated 99% confidence bounds. The results of the analysis using ERP data are shown in Fig. 2, where the error bars represent the 99% confidence bounds. Table 1 provides a detailed summary of the ERP features selected by the feature extraction approach. In this context, it must however be understood that while the selected features are confined to a single data/time point, electrode (and frequency in case of the TF-decomposed data), this does not necessarily mean that all other features are not predictive at all. Given that the EEG records a continuous physiological signal showing autocorrelations, it is indeed quite likely that data points, that are neighboring a predictive feature in terms of temporal, topographic, or frequency properties, are also predictive to a certain, albeit lesser extent.

**Table 1:** Summary of the extracted features showing feature number, electrode site, time point in ms of the extracted feature after stimulus presentation, the mean predictability and the significance as provided from the t-tests used as a filter method in the feature selection step.

Feature number	Electrode	Time point (ms)	Mean predictability	p-value
1	C3	322	68%	0.008
2	Cz	1102	70%	0.006
3	CP2	35	71%	0.014
4	CP3	66	73%	0.015
5	TP7	457	74%	0.013
6	CP2	320	74%	0.008
7	C1	1117	75%	0.01
8	CP3	55	75%	0.01
9	CP5	887	76%	0.006
10	CP2	914	76%	0.006
11	C1	301	77%	0.006
12	Cz	1105	78%	0.004
13	C2	74	79%	0.003
14	CP5	406	79%	0.003
15	CP1	320	79%	0.003
16	FC2	270	79%	0.003
17	FC4	367	79%	0.003
18	FC2	1262	79%	0.003
19	F7	98	79%	0.003
20	C2	70	79%	0.003

The first ERP-feature (322 ms after Nogo stimulus presentation at electrode C3) led to a prediction accuracy of ~68%, which is significantly different from chance level as indicated by the 99% confidence bounds. Adding more features led to a numerical increase in prediction accuracy (refer Fig. 2 and Table 1), but this increase was not statistically significant, since the 99% confidence bounds largely overlapped with the prediction accuracy obtained after adding the second feature. The sLORETA analysis shows that activation at the time point of this most predictive feature was located in the left motor cortex (M1) (precentral gyrus, BA4) (refer Fig. 2).

A described in the Materials and Methods section, we conducted an add-on analysis with separate training and validations sets in order to evaluate the generalization ability of the selected features. Table 2 provides information on the ERP features selected by the second analysis. Given that the accuracy of selected feature in the validation set ranges from 64% up to 69% (see third column in Table 2), we can assume that only the first feature is suitable for prediction. The best predictive feature that was selected in our ERP training set was at 340 ms at electrode C1. Importantly, the best predictive ERP features were extremely similar in both analysis, as they were detected (almost) in the same location (C3 and C1, which are directly neighboring electrodes) and time range (322 ms and 340 ms). Besides that, the accuracy for the best feature in the validation set for ERP data was 64% (i.e. it is above chance level and similar to what we observed without a separate validation set). Without this feature, the prediction accuracy drops to chance level (i.e. 50%), which further indicates the importance of this feature for the classification process. Finally, in 99% of our permutation tests, the feature’s the prediction accuracy of the real performance group (64%) was higher than the accuracy for randomly assign labels. This means that this feature was not selected by chance. As none of the classical ERP component peaks (such as N2 and P3) were selected by the algorithms, we additionally set out to assess whether and how much selected features are better in predicting inter-individual performance variation/group membership than the classical ERP components. We therefore compared the predictive value of these classical ERP components (as quantified for the classical ERP analysis) to that of the selected feature. To this end, the mean amplitudes of N2 and P3 (quantified in the time window from 250 ms to 280 ms at electrode Cz and from 370 ms to 410 ms at electrodes FC1 and P1) were entered into SVM in order to predict group membership. Of note, the prediction accuracy for these features was 46% and hence at chance level. This clearly demonstrates that conventional ERP components are not always the best features, or even useful for the prediction of behavioral performance. The reason why 46% is at chance level is a follows: There are N = 240 subjects available for the SVM analysis. We used the k-fold method to validate classifier performance. Since n = 10 this means that there are N = 24 subjects per cross-validation step. Consequently, the resolution for accuracy is 4.17% (1/24). Therefore, 46% is within the chance level range. To further evaluate whether this results is biased by the choice of the behavioral performance index, we performed another analysis in which classical ERP components were used as potential features for classifying good and bad performance on the basis of RT data and percentage of correct inhibition in the Nogo condition. Classification accuracy RT was 51% and 55% for the percentage of correct inhibitions in Nogo trials. This did not differ from chance level.

**Table 2:** Summary of the extracted features for the second analysis with training and validation sets for ERP data.

Number of features	Accuracy in training set	Accuracy in validation set	% in which the prediction is better than randomly assigned labels	Accuracy without selected features
1	72%	64%	99.9%	50%
2	76%	65%	99.8%	50%
3	79%	65%	99.7%	50%
4	79%	67%	99.9%	50%
5	80%	67%	100%	50%
6	81%	69%	100%	50%
7	82%	69%	99.9%	50%
8	83%	69%	100%	50%
9	83%	69%	100%	50%
10	83%	68%	100%	50%
11	83%	65%	99.9%	50%
12	83%	67%	99.9%	50%
13	83%	65%	99.8%	50%
14	83%	68%	99.9%	50%
15	83%	69%	99.8%	50%
16	83%	69%	100%	50%
17	83%	69%	100%	50%
18	84%	69%	100%	50%
19	83%	68%	99.9%	50%
20	84%	68%	100%	50%

All accuracy values are cumulative.

**Figure 3:**
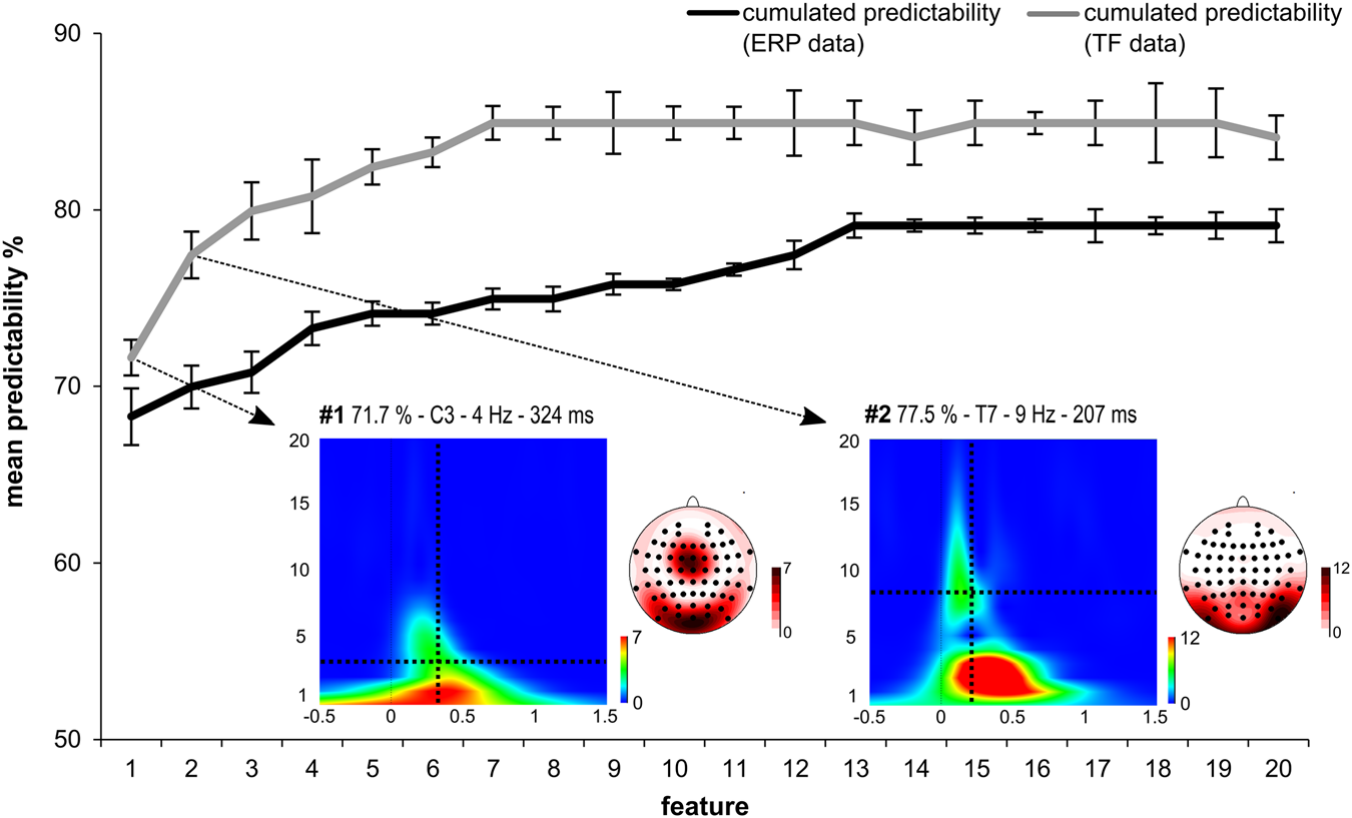
Results from the classification analysis using the TF-decomposed data (grey line). The mean predictability is given depending on the number of features. The grey curve in the figure shows the cumulative mean predictability. The error bars represent the 99% confidence level bounds. The black curve showing the cumulative predictability using ERP data is given for comparison. As can be seen the confidence bounds were not overlapping for the first and the second feature (grey curve). The TF plots of the first and the second feature are also shown. The dashed vertical lines in these plots show the time point/frequency (feature) at the respective electrode site (C3 for the first feature and T7 for the second feature). The scalp topography plots reveal the distribution of voltages for these features.

While the results on the basis of ERPs already show that behavioral performance can be predicted with an accuracy of up to ~68% with the help of our automatically selected feature, the results of the analysis using the TF-decomposed data show that prediction accuracy increased to ~78%. The results of the analysis using TF data in comparison to the ERP data are shown in Fig. 3. In Fig. 3 the error bars represent the 99% confidence bounds. Table 3 provides a detailed summary of the TF-features selected by the feature extraction approach. There were two TF-features which significantly increased predictability of behavioral performance. The first TF-feature (324 ms after Nogo stimulus presentation at electrode C3) revealed a predictability of behavioral performance of ~72% (refer Fig. 3 and Table 3) and was found in the theta frequency band (i.e. at 4 Hz). The 99% confidence bounds did not overlap between the first ERP-feature and the first TF-feature indicating that predictability of behavioral performance was significantly better using TF than ERP data, even though both were found almost at the same time point and at the same electrode. The same was the case when adding the second TF-feature (207 ms after Nogo stimulus presentation at electrode T7). The second TF-feature increased predictability to ~78% and was found in the alpha frequency bands (i.e. at 9 Hz). As can be seen in Fig. 3, the addition of further features numerically increased predictability, but this was not significant given the overlap of the 99% confidence bounds for each of the added features. Importantly, the predictability of the identified features was a little higher for the high performance group (72.45% for the ERP feature alone and 81.67% for the combination of ERP features and the two TF features) than for the low performance group (64.15% for the ERP feature alone and 73.30% for the combination of ERP features and the two TF features). Finally, it is essential to directly compare the classification results obtained using the ERP features and the TF features. Please refer to Tables 1 and 3. For the ERP data it is shown that the best accuracy than we can achieve is about 79% percent with 13 features. However, for TF analysis the highest accuracy is 85% and is achieved with 7 features. However, it needs to be stressed that including more than the best predictive ERP feature, and more than the first two (best predictive) TF-feature did not lead to a statistically significant increase in the classification accuracy. There, above-mentioned maximal prediction accuracy have to be treated with caution. If we just focus on the best feature for each analysis, the TF feature has a better classification accuracy (i.e. 72%) than the ERP-feature (i.e. 68%) (as indicated by non-overlapping confidence bounds).

**Table 3:** Summary of the extracted features showing feature number, electrode site, frequency, time point in ms of the extracted feature after stimulus presentation, the mean predictability and the significance as provided from the t-tests used as a filter method in the feature selection step.

Feature number	Electrode	Hz	time point (ms)	mean predictability	p-value
1	C3	4	324	72%	0.002
2	T7	9	207	78%	0.007
3	F1	7	109	80%	0.006
4	CP1	14	1078	81%	0.003
5	FC1	3	90	83%	0.006
6	CP1	15	1074	83%	0.006
7	C3	12	1023	85%	0.006
8	F4	6	90	85%	0.006
9	Fz	7	20	85%	0.005
10	F4	8	277	85%	0.004
11	FC3	13	684	85%	0.004
12	F4	6	94	85%	0.003
13	POz	14	63	85%	0.005
14	T8	8	121	84%	0.003
15	TP9	10	813	85%	0.004
16	CP3	13	996	85%	0.007
17	T8	7	125	85%	0.001
18	CPz	4	996	85%	0.005
19	C3	11	1023	85%	0.009
20	FC4	7	195	84%	0.001

A described in Materials and Methods section, we conducted an add-on analysis with separate training and validations sets in order to evaluate the generalization ability of the selected features. Table 4 provides information on the TF features selected by the second analysis. For TF data as described in the Table 4, the accuracy in the validation set ranges from 67% to 70%. We therefore only focused on the first feature. The best predictive feature occurred at 340 ms at electrode C3 in 4 Hz. Notably, the best features for TF data are very similar in both analyses (i.e. 324 ms and 340 ms at electrode C3 in 4 Hz). Moreover, prediction accuracy drops to 50% without this feature and in 98% of the permutation tests, the feature’s prediction accuracy was higher for the real performance groups than for randomly assigned labels.

**Table 4:** Summary of the extracted features for second analysis with training and validation sets for time frequency data showing feature number, accuracy in training set, accuracy in validation set, corresponding p-value for permutation test and accuracy without selected features.

Number of features	Accuracy in training set	Accuracy in validation set	% in which the prediction is better than randomly assigned labels	Accuracy without selected features
1	75%	67%	98.3%	50%
2	81%	70%	99.9%	50%
3	83%	70%	97.7%	50%
4	84%	70%	95.2%	50%
5	86%	70%	95.7%	50%
6	86%	70%	99.7%	50%
7	88%	69%	98.8%	50%
8	89%	69%	98.7%	50%
9	90%	69%	99.9%	50%
10	90%	69%	99.7%	50%
11	90%	69%	98.8%	50%
12	90%	70%	96.2%	50%
13	90%	69%	98.3%	50%
14	90%	70%	99.8 5	50%
15	90%	70%	100%	50%
16	90%	70%	99.9%	50%
17	90%	70%	99.7%	50%
18	90%	70%	100%	50%
19	90%	70%	100%	50%
20	90%	70%	100%	50%

All accuracy values are cumulative.

## Discussion

In the current study, we used a Go/Nogo task and a machine learning approach to predict inter-individual behavioral performance differences in response inhibition on the basis of neurophysiological data. We used a purely data-driven strategy to extract neurophysiological features that predict response inhibition performance, as assessed by performance group membership. In this context, it should be noted that even though our group formation criterion was relative (rather than absolute or categorial) it is the most appropriate criterion for our research question, i.e. the mechanisms underlying inter-individual performance variations. This focus was set to provide a rigorous test about the relevance of different neurophysiological features and associated functional neuroanatomical structures that are currently considered to best reflect inter-individual variation in inhibitory control. Importantly, we used both time-domain (ERP) and time-frequency decomposed ERP data from a sample of N = 240 subjects in an SVM approach as not all frequencies might be equally important for response inhibition. Additionally, such a data-driven procedure may allow to generate new testable hypotheses^40^ about the neurophysiological and functional neuroanatomical processes driving response inhibition processes.

An analysis of the standard ERP-components revealed the usual pattern of results^2^. The results from the SVM analysis showed that both time-domain data (ERPs) and time-frequency (TF) decomposed data collected in Nogo trials allowed to predict behavioral performance above chance level. Yet, predictability was significantly higher using TF-decomposed data (~78%) than when using ERP data (~68%). We had anticipated this effect as ERPs are a mixture of various oscillations, not all of which are (equally) important for the investigated cognitive process (Hoffmann *et al*.^21^).

For the ERP data, a single feature was shown to predict behavioral performance (i.e. group membership) with up to ~68%. While the Nogo-N2 and Nogo-P3 peaks usually constitute the neurophysiological processes that are usually focused on in inhibition research (for review^2^), the predictive feature was identified in-between these two ERPs, i.e. in the transition between these two processes. Moreover, the neuroanatomical source of the feature differed from that of the two ERPs: At the time point of the predictive feature, the sLORETA analysis suggested that left hemisphere motor areas (precentral gyrus, BA4) were most strongly activated (refer Fig. 2). In line with previous work^2,43–46^, the sLORETA analysis however revealed medial frontal areas including the superior frontal gyrus (BA6) and regions in the anterior cingulate cortex (ACC, BA24) to be most strongly activated at the time point of the Nogo-N2 and P3 (refer Fig. 1). Interestingly, the frontal-midline Nogo-N2 component has been suggested to reflect pre-motor processes like conflict monitoring or updating of the response program. In this context, the finding that the predictive feature occurred after the Nogo-N2 and was associated with an activation of the motor cortex (precentral gyrus) seems reasonable and allows for the interpretation that the predictive ERP feature reflects mechanisms where pre-motor processes are transferred to motor programs in order to inhibit the response. This interpretation is corroborated by the finding that responses had to be executed with the right hand / left motor cortex and that the time point of the predictive feature (~322 ms) was before the mean RT (~350 ms). fMRI studies have suggested that the pre-supplementary motor area and the motor cortex are involved in inhibitory control^27,47^. Furthermore, both of these structures show functional interconnectivity via the basal ganglia circuitry that mediates response inhibition. As mentioned, the predictive feature occurred before the Nogo-P3 peak (~405 ms), which has been suggested to reflect evaluative processing of the successful outcome of the inhibition^2,15–17,48,49^ and is thus thought to reflect the cognitive process of assessing whether (not) responding was correct^2,50,51^. This interpretation was mainly based on the assumption that the Nogo-P3 peaks too late (i.e. after the mean RT) to reflect motor inhibition processes^52,53^ and has been criticized in the past^18^. The fact that the predictive feature was found well before the Nogo-P3 and seems to be related to the motor process however supports the interpretation of evaluative processes being reflected in the Nogo-P3. Even though the Nogo-N2 and Nogo-P3 reflect sub-processes involved in response inhibition^2^, machine learning analysis using ERP data that they are not necessarily important to consider when trying to predict the behavioral outcome of response inhibition, as only the time window in between both ERPs could predict performance. This was furthermore underpinned by the finding that neither the N2, nor the P3 amplitudes could predict performance (i.e. classify group membership) above chance level. While this does not necessarily refute the theory that the Nogo-N2-P3 complex reflects sub-processes of inhibitory control, the results suggest that processes which take part in the transition between both ERPs, should receive much more attention.

Interestingly, the machine learning results obtained for the time-frequency decomposed data revealed a feature at the same electrode (C3) and almost at the same time point (i.e. 324 ms) within the theta frequency band. The convergence of results (i.e. time point and electrode) between ERP and TF-decomposed data further underlines the reliability of the obtained data. In this context, it however needs to be noted that while our predictors are single time points, they are not necessarily isolated events which solely predict group membership. Instead, they are an integral part of cognitive processes, which should be interpreted as a whole for a better understanding of the mechanisms driving response inhibition. Several lines of evidence have already shown that response inhibition processes reflected by the Nogo-N2-P3 complex are associated with oscillations in the theta frequency band^2,15,29–32^. It is therefore plausible that a feature in the theta frequency band was predictive of behavioral performance. This finding is also well in line with literature suggesting that oscillations in the theta frequency band are particularly important for cognitive control and executive functions^22–26^. But while the results showed that the theta frequency feature was most predictive, predictability significantly increased from ~72% to ~78% when additionally considering a second feature in the alpha frequency band ~207 ms after Nogo stimulus presentation. The alpha frequency band has been suggested to be important for inhibitory control^33,36,37^, as oscillations in this frequency band coordinate top-down control processes^34,38^. Specifically, alpha oscillations have been suggested to reflect inhibitory mechanisms that control access to task-relevant information;^36^ i.e. updating processes of internal task sets being used for other cognitive processes^35^. Since the predictive alpha band feature was detected at ~207 ms, it was evident in the time window of the P2 ERP component. The P2 has been suggested to reflect resource allocation processes^54–56^. Such processes are closely related to mechanisms which are necessary to update internal task sets and hence processes that are considered to be reflected by alpha oscillations. There were no linear correlations between electrophysiological parameters and behavioral performance, which may be due to the fact that regression approaches and correlations can only model linear interdependencies. This, however, is not the case using SVM approaches.

As the results from the machine learning (SVM) approach were obtained using a data-driven procedure, they call for a change in how we currently conceptualize and investigate response inhibition sub-processes: While theta oscillations and related processes reflected by the Nogo-N2-P3 complex may represent mechanisms operating during the process of inhibitory control^2^, neither the Nogo-N2 nor the Nogo-P3 peak are central to predicting behavioral performance. Rather, it seems that motor cortex-driven processes which take part in the transition between the two ERP peaks are most important for inter-individual differences in response inhibition performance. Currently, most studies have only considered the relevance of *either* theta *or* alpha oscillations for inhibitory control processes. The current results however show that the combination of frequencies best predicts inhibitory control performance and further suggest that the updating of internal task sets should probably receive more attention. Given that the Nogo-N2-P3 complex is found in a wide variety of inhibitory tasks, we would deem it likely that the obtained results are not only valid for Go/Nogo paradigms, but also for other paradigms (e.g. stop-signal tasks). In Go/Nogo paradigms, participants are required to inhibit a prepotent response tendency, whereas participants are asked to stop an requested a motor response in the SST. The latter increases the probability of an initial activation of the (Go) motor response process^49^. Even though both paradigms differ in the exact nature of the inhibitory processes e.g^57^^.^, it is generally assumed that both paradigms rely on a similar inhibitory control network^58,59^ and are associated with similar electrophysiological correlates^49^.

Regarding the generalizability of the current findings, it however needs to be noted that there are many other machine learning algorithms that can be used for feature extraction and prediction. It is hence possible that results might change when other algorithms/models are used. But in the words of George Box, it can be stated that „*Essentially*, *all models are wrong*, *but some are useful*“. Against this background, the usefulness of our results remains to be evaluated in future studies. That is, the functional relevance of the identified processes and functional neuroanatomical structures should be investigated in more detail in the future. However, it needs to be noted that there is (i) accumulating evidence reporting an important role of the motor cortex, but not medial prefrontal areas for response inhibition (see above) and (ii) the entire pattern of results using ERP and TF-decomposed data is conclusive. It may seem arbitrary that subjects were classified by splitting the group into “good” vs. “bad” performers, but this is a necessary step to be able to apply the SVM approach. Yet, it does not necessarily describe the range of individual differences in task performance. Thus, the data may more strongly support the idea that these alternative approaches may extract features that are better predictive of relative task performance. The detection of novel neurophysiological features are possible important to consider in future studies, rather than specifically addressing variability in inter-individual differences.

In summary, our results suggest that when investigating response inhibition, we should not only focus on control-associated ERPs/oscillations and associated prefrontal cortical structures commonly considered to the reflect an important response inhibition network^27^. Instead, we should extend our focus to the motor cortex, theta *and* alpha frequency oscillations, and the transition between the classical Nogo-N2 and Nogo-P3 correlates of inhibitory control. Using brain stimulation techniques (e.g. TMS or tACS), it would be possible to directly test the importance of the neurophysiological processes identified in the current study.

## Materials and Methods

### Participants

A sample of N = 251 healthy subjects between 20 and 30 years of age (mean age 23.4 SD = 2.94; 99 females) was recruited for the study. None of the participants reported a history of neurological or mental illness. N = 11 participants were excluded from analysis due to poor performance (as defined by a mean subject’s Go RT > mean group Go RT + 3 SD, false alarms and/or misses ≥ 50%) or noisy EEG signal (visual inspection: when blinking or other artifacts could not be sufficiently removed with the help of an ICA). The data analysis was performed with N = 240 subjects. Of note, the participants/datasets included in this study have also been included in a previous publication^60^. Yet, we used a different behavioral measure for classification that circumvents the speed-accuracy issues we had in our previous publication, applied stricter exclusion criteria based on the EEG quality and used a vastly different methodological approach which did not limit the number of features to a rather small, theory-driven pre-defined set of ERPs^60^, but instead used the full range of available data. The study and all experimental procedures were approved by the ethics committee of the Ruhr Universität Bochum and TU Dresden. The methods were carried out in accordance with these regulations. Written informed consent was obtained from all participants.

### Task and behavioral parameter for group formation

We used a standard Go/Nogo task^16,31^, in which two stimuli were presented. Upon presentation of the German word “DRÜCK” (English “press”), the subjects were asked to respond as fast as possible. Upon presentation of the German word “STOPP” (English “stop”), the participants were asked to refrain from responding. The stimuli were presented in the middle of a 17inch CRT screen (100 Hz vertical refresh rate). The ratio of Go and Nogo trials was 70:30. This ratio ensures that there is a strong tendency to respond on Nogo trials making it difficult to refrain from responding upon the presentation of a Nogo stimulus. Subjects were asked to respond within 600 ms. If this response deadline was missed, the word “SCHNELLER” (English “faster”) was presented. This further augments the tendency to respond on Nogo trials. Each trial began with the presentation of a fixation cross in the middle of the screen and was terminated by the participant’s first response (correct responses in Go trials or false alarms in Nogo trials). In case of missed Go responses or a correct inhibition of responses in Nogo trials, the trial automatically terminated after 2200 ms had elapsed. The inter-trial interval (ITI) was jittered between 1000 and 1300 ms. On Go trials, responses exceeding this deadline were regarded as “missed” trials. In total, 450 trials were presented. As a performance measure in Go/Nogo tasks, the false alarm rate (i.e. responses upon the presentation of Nogo stimuli) is considered to be most important. However, the likelihood to commit a false alarm also depends on the speed of responding, with a higher response speed leading to higher false alarms rates^39^. To account for this, we calculated an index of false alarms in Nogo trials and the mean reaction time (RT) in Go trials; i.e. the percentage of correct inhibition in the Nogo condition (i.e. 100% minus false alarm rate) divided by mean hit RTs in the Go condition. Importantly, better performance may be reflected by better accuracy (i.e. larger accuracy values) and/or faster responses (i.e. smaller response time values). Based on this ratio, we used a median-split to form two equally large groups. Participants yielding relatively small ratio values were labeled “bad performers” while participants yielding relatively large values were labeled “good performers”. This performance index was used in further analyses using machine learning approaches; i.e. it was examined in how far performance group membership was predictable on the basis of neurophysiological features extracted from the neurophysiological data.

### EEG recording and analysis

The EEG was recorded from 64 Ag/AgCl electrodes using the extended 10/20 system against a reference electrode placed at electrode FCz (QuickAmp, Brain Products Inc.). The signal was automatically re-referenced to a common average reference by the amplifier. The sampling rate was 1 kHz. Electrode impedances were kept below 5 kΩ. After recording, the data were down-sampled to 256 Hz. The EEG was digitally filtered off-line using IIR band-width filters at 0.5 and 18 Hz (each with a slope of 48 dB/oct) using the BrainVision Analyzer 2 software package (BrainProducts, Inc.). Then, gross artifacts were manually removed from the EEG. Following this step, horizontal and vertical eye-movements as well as pulse artifacts were detected using independent component analysis (ICA) (infomax algorithm), and components reflecting these artifacts were removed. After reconstructing the EEG from the remaining components, electrode FCz was interpolated using a spherical spline interpolation. Then, the EEG was segmented into Go and Nogo trials. Only correct Go and Nogo trials were included in the data analysis. Only Go trials where the correct response was carried out within 1200 ms after stimulus onset were considered as correct. Nogo trials were included when no response was given in an interval of 2200 ms after stimulus presentation. The segments were 4000 ms long, starting 2000 ms before the locking time point (time point zero) and ended 2000 ms after this locking time point. Though this leads to an overlap between succeeding trials, this long epoch is needed for the time-frequency decomposition step to allow a reliable assessment of the power in low frequency bands^61^. On average, 287.7 (±8.8) Go trials and 106.9 (±12.9) Nogo trials were included. This segmentation step was followed by an automated artifact rejection procedure to eliminate any artifacts that might have survived the prior data inspection. A maximal value difference above 200 μV in a 100 ms interval as well as an activity below 0.5 μV in a 200 ms period were used as rejection criteria. About 2.7% of the trials were rejected due to artifacts. Next, a current source density (CSD) transformation^62^ (order of splines m = 4, maximum degree of the Legendre polynomials n = 10, precision of 2.72^−7^) was applied to re-reference the data. Due to this, the resulting CSD values are given in μV/m^2^. Baseline correction was applied in the time range from −200 ms to 0 ms (i.e. prior to target onset) before the segments were separately averaged for Go and Nogo on a single subject level to derive the event-related potential (ERP). All ERP components were quantified using local maximum peak detection on single subject level. For P1, a time range of 90 to 110 ms at electrodes PO9 and PO10 was used. For N1, the time range was set to 170 to 190 ms at electrodes PO9 and PO10. N2 ERP mean amplitudes were determined in the time range from 250 to 280 ms at electrode Cz. For the P3, the mean amplitudes were quantified from 370 to 410 ms at electrodes FC1 and P1. All electrodes were chosen based on scalp topography of each ERP component and condition. This procedure was validates as described by Mückschel *et al*.^63^ A time window of maximum activity was identified for each component. Using these time windows, the mean amplitude was extracted for every electrode. Each electrode was then compared against the average of all other electrodes using Bonferroni correction (p = 0.0007). Only electrodes with significantly larger amplitudes than the remaining electrodes were selected. In addition to this time-domain analysis of the data, a time–frequency analysis was conducted by means of a continuous wavelet transformation (CWT) employing Morlet wavelets ($w$) in the time domain to different frequencies (ƒ):

$$W(t,f)=A\,\exp (-{t}^{2}/{{\sigma }^{2}}_{t})\exp (2i\pi ft)$$

where *t* = time, *A* = (*σ*_*t*_ $\sqrt{{\rm{\pi }}}$)^−1*/*2^, *σ*_t_ = wavelet duration, and $i=\sqrt{-1}$. For analysis and TF-plots, a ratio of ƒ_0_/*σ*_ƒ_ = 5.5 was used_,_ where *σ*_ƒ_ is the width of the Gaussian shape in the frequency domain and ƒ_0_ is the central frequency. The analysis was conducted in the frequency range from 1 to 18 Hz, and a central frequency at 1 Hz intervals was employed. For different ƒ_0_, time and frequency resolutions [or wavelet duration and spectral bandwidth] can be calcula_*t*_ed as 2*σ*_*t*_ and 2*σ*_ƒ_ respec_*t*_ively. *σ*_*t*_ and *σ*_ƒ_ are related by the equation *σ*_*t*_ = 1/(2*πσ*_ƒ)_. For example, for ƒ_0_ = 1 Hz, 2*σ*_*t*_ = 1770 ms and 2*σ*_ƒ_ = 0.36 Hz; for ƒ_0_ = 3 Hz, 2*σ*_*t*_ = 580 ms and 2*σ*_ƒ_ = 1.09 Hz; for ƒ_0_ = 5 Hz, 2*σ*_*t*_ = 350 ms and 2*σ*_ƒ_ = 1.82 Hz. The total power was calculated by performing TF decomposition on the single trial level before averaging.

### Data-driven feature extraction procedure and support vector machine (SVM) analysis

Based on a median split of the behavioral performance parameter (i.e. correct inhibition rate on Nogo trials, relative to the RT on Go trials), two groups of subjects were created: a “high performance group” and a “low performance group”. Thereafter, a machine learning approach was employed to predict group membership on the basis of the neurophysiological data from Nogo trials. Only neurophysiological data from correct Nogo trials, i.e. trials where no response was executed, was used.

For the time-domain features, all time points from zero (i.e. locking time point/time point of stimulus presentation) to 1.5 seconds with the resolution of the sampling frequency (256 Hz) were extracted as possible features. This was done for each of the 64 channels and for every subject. For the time-frequency decomposed data the same approach was applied for every frequency in the frequency range between 1 and 18 Hz using 1 Hz frequency steps. Moreover, before using feature selection and classification methods, and in order to eliminate the effect of different range of features on classification performance all features were normalized into a z-score. This was done for two reasons: First, it increases the convergence speed of feature detection algorithms^64^. Second, features may govern/bias the feature detection algorithm in case they have different value ranges. This problem is circumvented using z-transformation^65^, because z-transformation makes all features have a mean of zero and a standard deviation equal to one. After normalizing the features, the feature selection was applied. This is crucial for machine learning algorithms, since it eliminates surplus/irrelevant features and reduces the problem of having a ‘small’ data set relative to the size of the possible feature set. Both of these factors can otherwise reduce the classifier performance. In the feature selection stage, an optimal subset of features is selected from the original feature set. Feature selection algorithms roughly divide into two categories: “filter” and “wrapper” methods^66^. The filter methods select a subset of features according to general characteristics of the data, independently of chosen classifier. Wrapper methods require a predetermined classifier and evaluate features according to their performance to discriminate between classes^66^. Usually, wrapper methods lead to better results than filter methods, because the selected features are based on classifier performance^67^. However, they can be significantly slower than filter methods. One way to overcome this problem is to combine filter and wrapper methods: i.e. filter methods are applied first to select some features. Then, these selected features are used as input for wrapper methods. This was done in the current study using MATLAB 2017a (Mathworks Inc.). In particular, t-test and sequential floating forward selection (SFFS) methods are employed as a filter and wrapper methods, respectively^67^. For initial sorting of the features, a t-test is calculated between the two groups created using the median split procedure for each time point (i.e. feature). If the p-value is below 0.01 this time point (feature) is selected (the precise p-values are given in Table 1 and Table 3 in the results section). Then these selected features are used as input for the SFFS algorithm. SFFS combines two separate algorithms;^68,69^ sequential forward selection (SFS) and sequential backward selection (SBS). SFS starts from an empty set of features and sequentially adds features that result in the highest classifier accuracy when being combined with the features that have already been selected. SBS works in the opposite direction. In SFFS, each feature selection step comprises SFS and SBF^68,69^ and were implemented in MATLAB 2017a (Mathworks Inc.). Following SFFS, selected features are fed to a support vector machine (SVM) employing a radial basis function (RBF) kernel with sigma equals to 5. This was done using MATLAB 2017a (Mathworks Inc.) and the LIBSVM toolbox^70^. SVMs are supervised learning algorithms that aim towards an optimal separation of distinct classes. In other words, the applied classifier projects input data into high-dimensional feature space to determine a hyper-plane which is able to separate the groups.

Importantly, the result of the SVM method was cross-validated in this study. Since neuroimaging studies usually deal with a ‘small’ number of subjects, it is very important to use cross-validation methods. There are two popular methods that can be used: Leave One Out Cross Validation (LOOCV) and k-fold cross-validation^71^. However, the high variance of the classification accuracy and the computation time are two major problems of the LOOCV method^72^. The k-fold method randomly divides the data into k portions in which k-1 portion is considered as training data and other as testing data. By continuing this k-times all subjects in the data set are part of the testing and training set. The resulting classification accuracy is the average of the all k-folds^71^. Usually, the value of k is between 5 to 10 in machine learning. We used k = 10 in this study. This means that for each extracted feature there were 10 estimations of the predictability of behavioral performance. Using the data from the k = 10 estimations we calculated the 99% confidence bounds for each feature. These confidence bounds were then used to examine (i) in how far the different features provided a significant increase predictability of behavioral performance, and (ii) in how far there is a difference in the predictability of behavioral performance between ERP and TF-decomposed data. A significant difference is indicated by no overlap between the calculated 99% confidence bounds. Importantly, the applied machine learning approach also minimizes the risk of ending up with false positive features: While there is indeed still a small risk for false positive features to survive feature selection and enter the machine learning approach, the subsequent k-fold validation procedure, which is an integral part of the machine learning approach, minimized the risk of any false positive being ultimately selected as a predictive feature because it mixes and recombines the sample many times, which strongly decreases the likelihood of false positives having a strong and consistent effect.

Since the feature selection and evaluation steps were done in the same data set, it could be argued that the generalization ability (i.e. working well on other data sets) required further verification. In order to demonstrate the validity of the initially applied k-fold method and verify that the selected features do not have an overfitting problem (i.e. that they do not work well only on the training set), we ran an additional analysis, for which the data were divided into two separate data sets (training and validation). 70% of the subjects were used as the training set (168 subjects) and the remaining subjects (72) as the validation set. In the training dataset, we again employed the k- fold and feature selection methods (t-test and SFFS) in order to achieve maximal comparability. For validation of the results (i.e., the features that had been selected in the first data set), we used SVM in the validation set. In order to have a comprehensive assessment of selected features, we also used permutation tests for the features selected from the training set and applied it on the validation set. For those permutation tests, the data were randomly divided into two groups and SVM was used for the prediction of those random groups. This process was repeated 1000 times. In the end, we calculated the percentage of how many times (out of 1000) the selected feature from the training set predicted group membership better than a randomly assigned group label. To confirm that the selected features are practically important for classification accuracy (i.e. to demonstrate that the accuracy will drop to chance level without them) we investigated the impact of omitting these features on classification accuracy. To this end, we started to consecutively omit the best features and ran the SVM without them. In this context, it is worth mentioning that we trained the classifier on the training set and the results are just based on the validation set.

### Source localization analysis

For each of the time-domain (ERP) features which were shown to be predictive for behavioral performance in the SVM analysis step (see results section) a source localization analysis was conducted. For this analysis, sLORETA (standardized low resolution brain electromagnetic tomography;^73^ was used, which provides a single solution to the inverse problem^73,74^. For sLORETA, the intracerebral volume is partitioned into 6239 voxels at 5 mm spatial resolution. Then, the standardized current density at each voxel is calculated in a realistic head based on the MNI152 template. It has been mathematically proven that sLORETA provides reliable results without a localization bias^75^. Moreover, there is evidence from EEG/fMRI and neuronavigated EEG/TMS studies underlining the validity of the sources estimated using sLORETA^21,76^. The voxel-based sLORETA images were compared against zero using the sLORETA-built-in one sample t-test. Importantly, voxel-wise randomization tests with 2000 permutations, based on statistical nonparametric mapping (SnPM) were applied to control the risk of false positive results. Significant voxels (p < 0.01, corrected for multiple comparisons) were located in the MNI-brain.

### Statistics

A standard analysis of the electrophysiological data was performed using repeated measures ANOVAs including the within-subject factors “condition” (Go vs. Nogo trials) and “electrode” as well as the between-subject factor group (low vs high performance) in the model. Greenhouse-Geisser correction was applied wherever appropriate and all post-hoc tests were Bonferroni corrected. As indicated by Shapiro-Wilks tests and confirmed by visual inspection, all analyzed variables were normally distributed (all W > 0.51; df = 239; p > 0.212). For the descriptive statistics, the mean and standard error of the mean are given.

## Electronic supplementary material

Supplemental material

## Data Availability

The datasets generated during and/or analysed during the current study are available from the corresponding author on reasonable request.
