# Supplementary material for: Machine learning provides novel neurophysiological features that predict performance to inhibit automated responses
Source: Sci Rep. 2018 Nov 2;8:16235. doi: 10.1038/s41598-018-34727-7 (PMC6215005; doi:10.1038/s41598-018-34727-7)
Supplement: Supplementary file 1 — Supplemental material [file 41598_2018_34727_MOESM1_ESM.docx]

**Supplemental Material**

**Machine learning provides novel neurophysiological features that predict performance to inhibit automated responses**

Amirali Vahid, Moritz Mückschel, Andres Neuhaus, Ann-Kathrin Stock, Christian Beste

**ERP plots by group**

**
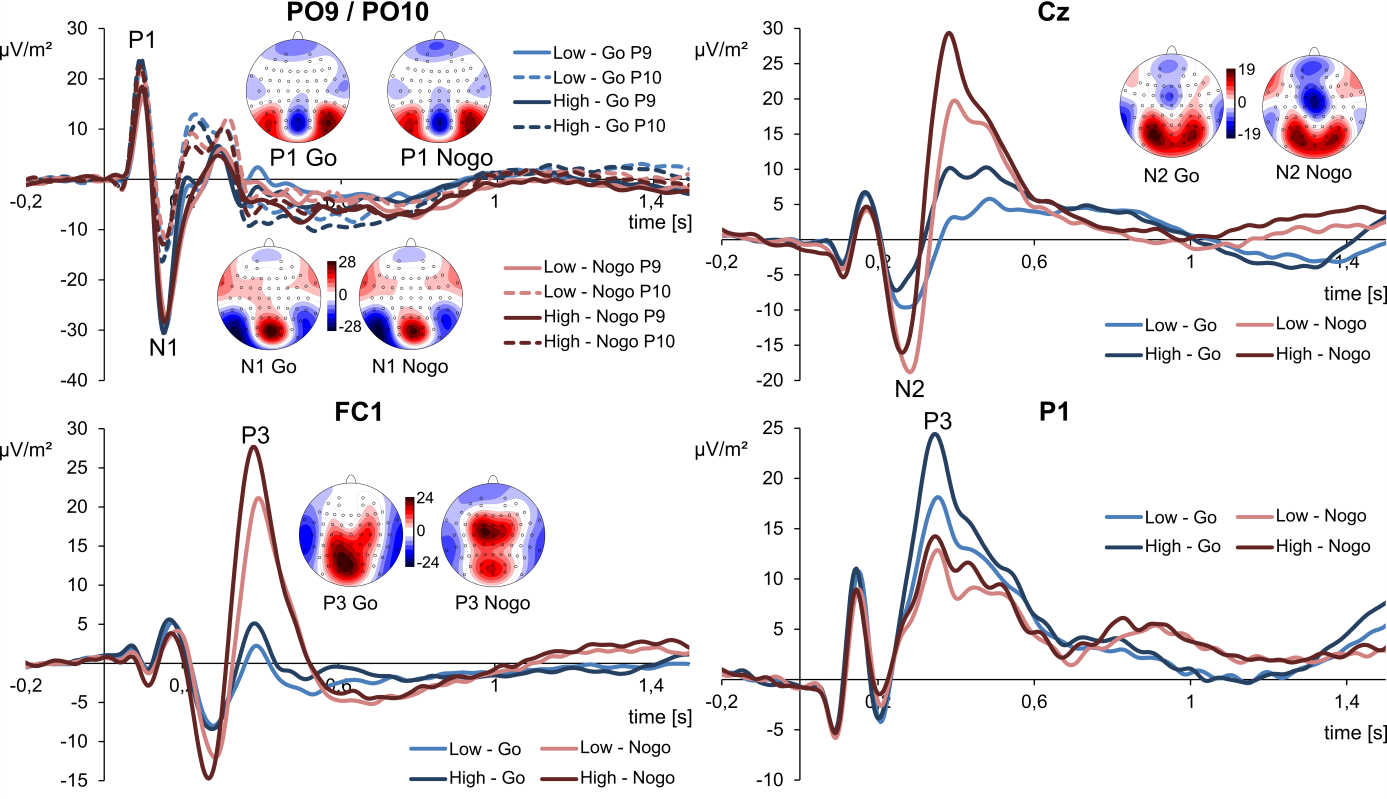
**

Supplementary Figure 1: Event-related potential (ERP) components on Go and Nogo stimuli presentations for low and high performance group. Plots are given for electrodes PO9/PO10 depicting P1 and N1 ERP, electrode Cz for N2, electrode FC1 for Nogo-P3 as well as electrode P1 for Go-P3. ERPs on Go stimuli are shown in blue, ERPs on Nogo stimuli are shown in red. The scalp topography plots reveal the distribution of voltages at the time point of the peak maximum of each ERP component. Time point zero denotes the time point of stimulus delivery.

**Time frequency analysis of alpha band activity**

As can be seen in supplemental figure for Nogo trials (right), a power increase is evident in the theta frequency band about 200 to 350 ms post stimulus with a fronto-central topography. To analyze whether this power increase differed between conditions as well as between groups, a repeated measures ANOVA (condition x groups) was calculated. Therefore, the mean theta oscillatory power was determined in the frequency range of 5 to 6 Hz, in the time window of 240 to 320 ms post stimulus for pooled electrodes FCz and Cz. There was a significant main effect of condition (F1,238) = 192.51; p < .001; η²_p_ = .45). Theta power was larger for Nogo trials (87.35 ± 4.70) than for Go trials (33.00 ± 1.55). All other effects were not significant (all F < 2.84, p > .093).

Supplementary Figure 2: Time frequency plots showing electrode CZ for Go and Nogo trials.


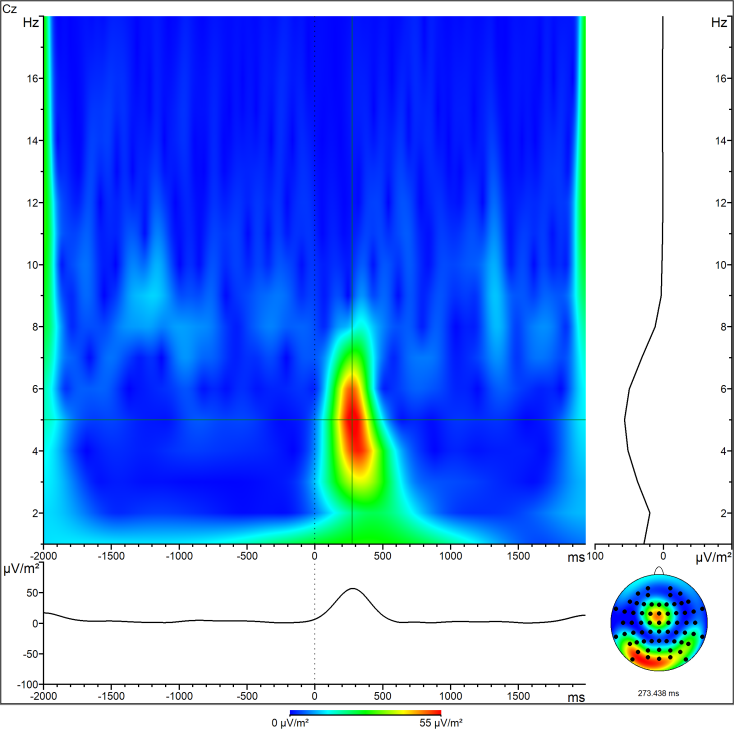

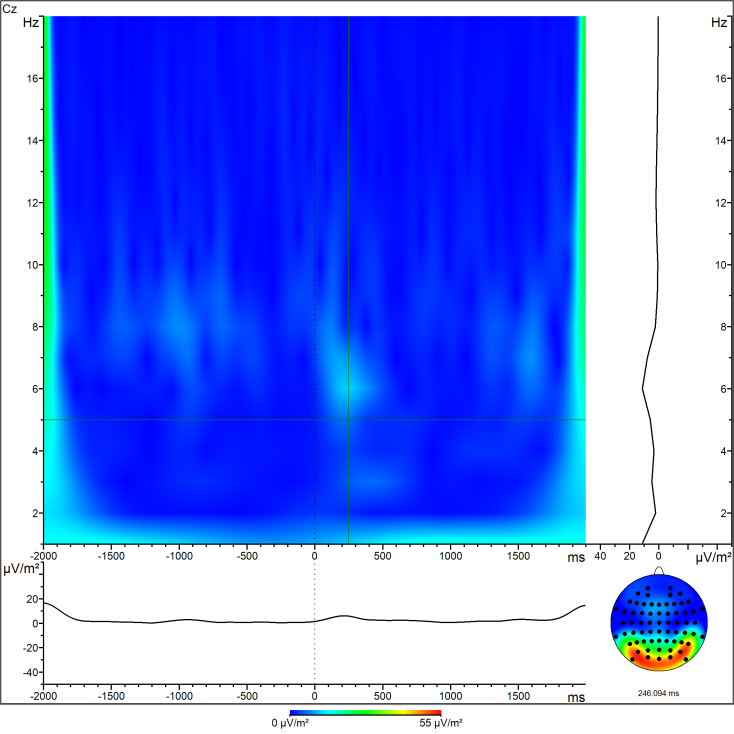


**Cz - Go**

**Cz - Nogo**

**Performance index**

**
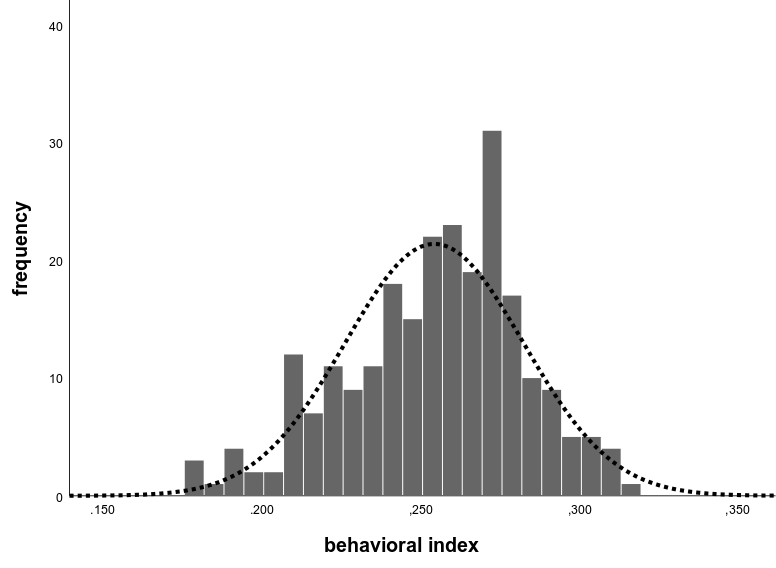
**

Supplementary Figure 3: Distribution plot of the behavioral performance index.
